# Critical care nurses’ and physicians’ knowledge, attitudes, and self-reported behaviors in mobilizing older adult patients: implications for optimizing geriatric care

**DOI:** 10.1186/s12912-025-03696-4

**Published:** 2025-09-18

**Authors:** Shimmaa Mohamed Elsayed, Asmaa Mahmoud Ali Ibrahim, Sameer A. Alkubati, Mohamed Hussein Ramadan Atta, Heba Hashem Monged

**Affiliations:** 1 Critical Care and Emergency Nursing Department, Faculty of Nursing, Damanhour University, Damanhour City, Egypt; 2 Gerontological Nursing Department, Faculty of Nursing, Alexandria University, Alexandria City, Egypt; 3 Department of Medical-Surgical Nursing, University of Hail, Hail, Saudi Arabia; 4 Nursing Department, College of Applied Medical Sciences, Prince Sattam Bin Abdulaziz University, Wadi Addawasir, Saudi Arabia; 5 Psychiatric and Mental Health Nursing Department, Faculty of Nursing, Alexandria University, Alexandria City, Egypt; 6 Critical Care and Emergency Nursing Department, Faculty of Nursing, Alexandria University, Alexandria City, Egypt

**Keywords:** Barriers, Critical care nurses, Older adult patients, Mobility, Physicians, Intensive care units

## Abstract

**Purpose:**

This study aimed to identify knowledge, attitudes, and behaviors regarding older adult patients’ mobilization from the perspectives of critical care nurses (CCNs) and physicians.

**Methods:**

The researchers employed a cross-sectional study design following the STROBE guidelines. A total of 136 CCNs and 64 physicians completed an online electronic survey. This single-center study utilized the PMABS-ICU questionnaire and adopted a convenience sampling method.

**Results:**

Two hundred voluntary participants were involved in our study. The mean (SD) of the overall barrier score for CCNs was 113.16(16.7), significantly higher than the 107.75(10.9) reported by physicians (*P* = 0.007). The mean (SD) attitude in CCNs was 26.75(5.00), which was of higher significance (*p* = 0.000) than physicians at 23.56(4.78). CCNs had a higher total mean (SD) behavior subscale, 45.22(11.20), than physicians, 44.39(4.79), with no statistically significant differences between them (*p* = 0.461). There was a moderate positive correlation between the overall score and subscale knowledge (*p* = 0.000), attitude (*p* = 0.000), and behavior (*p* = 0.000).

**Conclusion:**

CCNs reported that barriers to older adult patients’ mobility were perceived as higher than physicians. Safety fears and anticipation regarding falling can hinder CCNs’ initial attempts to promote the mobility of older adult patients, while physicians perceive time constraints as a hurdle.

**Implications for practice:**

Mobility barriers among older adult patients in ICUs were reported to be higher among CCNs than physicians, with fear of injury and safety concerns being the most significant barriers.

**Clinical trial number:**

Not applicable.

## Introduction

Older adults are a growing demographic in high-income countries, accounting for an increasing amount of health expenditure [1]. There will be 1.5 billion older adults, twice the current number, by 2050 [2]. The older adult population in Egypt is expected to increase to 20.8% by 2025. They comprise a growing percentage of patients admitted to intensive care units (ICUs) [3]. Caring for older adult patients in the intensive care unit is essentially different from caring for younger patients since it requires knowledge and skills. The prevalence of comorbidities, defined as the coexistence of many chronic diseases, tends to escalate with advancing age [4]. Multimorbidity, malnutrition, bedsores, healthcare-related infections, and inpatient mortality were associated with lengthy hospital stays and increased risk of falling and fractures [5, 6]. Additionally, the aging process, especially muscle weakness, affects older adults and may be associated with a decline in their physical and cognitive function, quality of life, and hospital admission of older adult patients [7]. 

Early mobilization is any movement outside of the patient’s range of motion (ROM) within 48 h following the start of mechanical ventilation, from passive to active mobilization [8]. Mobilization programs such as Function Focused Care for Acute Care and nurse-driven mobility intervention (NDMI) are complex for healthcare providers with training and a multidisciplinary team [9, 10]. Jamshed and Gangavati [11] mention mobility as a “5 M” holistic framework approach to care for older adult patients. Mobility issues associated with aging can lead to declining independence, quality of life following a critical illness, and the necessity for continuous high-level medical and nursing care. The worldwide prevalence of falls among the older adult population was 26.5% [12]. The incidence rate of falls in ICUs ranged from eighteen per 1000 bed days [13]. Therefore, older adult patients’ mobility in ICUs may be restricted because they are more likely to fall and have other adverse outcomes. Daily mobility for older adult patients in ICUs decreased hospital stays by 5% [4]. 

Most studies focus on investigating barriers and facilitators of mobilizing critically ill patients. A study was conducted by Crooks et al. [14] to identify knowledge, attitudes, or behaviors associated with barriers to mobilization among critical care nurses (CCNs) and physical therapists. Physiotherapists and CCNs face obstacles in patient movement, particularly among nursing personnel, requiring further research and collaboration between treatment and nursing providers. Another study done by Babazadeh et al. (2021) reported that more than half (76.6%) of the nurses had inadequate mobility training; 74% considered a shortage of nursing staff as a barrier to early mobilization; 57.9% of nurses reported a lack of time; and the majority of nurses considered patient-related obstacles to be the most challenging, such as coma, obesity, agitation, and being in pain [4]. 

The perspective of CCNs and other health care providers (physicians, nurses, aides, physiotherapists) toward older adult patients’ mobility needs requires further investigation. This may be due to older adult patients in ICUs being challenged with applications of mobility activities, as reported unfavored attitudes for nurses in studies conducted in different countries [2, 15–18]. Several studies reported that the primary obstacles to adopting mobility for older adult patients are insufficient training, lack of knowledge, and a negative attitude toward caring for older adult patients [19–21]. 

Despite their benefits, mobility activities are not frequently used in the ICU, with rates of 19.2% among ventilatory support patients and 23.5% for non-ventilatory support patients [22]. Integrating physical activity promotion and environmental adjustments can significantly improve the quality of life for older adults with older syndromes, requiring investigating healthcare providers’ inner perspectives to cater to this growing population’s unique needs effectively [23]. 

## Methods

### Aim of the study

This study aims to identify knowledge, attitudes, and self-reported behaviors regarding older adult patients’ mobilization among CCNs and physicians. The authors refer to mobilization as getting out of bed or sitting on the edge of the bed with or without mechanical aids.

### Study design

A descriptive cross-sectional research study design was used to comply with the STROBE statement [24]. 

### Settings

The ICUs of Alexandria Main University Hospital, serving a significant portion of the Alexandria governorate’s population, conducted the current study. The total capacity of beds is nearly 50 beds divided into five units. The capacity of the beds in each unit is ten beds. Each bed is equipped with a mechanical ventilator and a cardiac monitor. The nurse-patient ratio is 1:2. The ratio of admission of older adult patients aged over 60 years to patients aged less than 60 in this unit has been around 3:1 in the last seven months. The previous setting admits older adult patients with multiple disorders, necessitating close monitoring and high-quality care. In these units, mobility activities include sitting on a chair or walking.

### Participants

The authors collected the data using a convenience sampling technique. The previously mentioned setting had a total population of four hundred CCNs and physicians. The maximum sample size was calculated using Epi Info 7.2.4.0, and it was 197, guided by the formula Sample size n = [DEFF*Np(1-p)]/[(d2/Z21-α/2*(N-1) + p*(1-p)] where Z is the critical value and n is the sample size. The study involved all nurses (*n* = 136) and physicians (*n* = 64). CCNs affiliated with the faculty of nursing and physicians were invited to the study. The response rate was 95%. We conducted an online survey targeting staff members in the previously selected units from March 30th to November 30th, 2023. The study excluded participants with no previous experience caring for older adult patients and those with less than one year of experience. We recruited participants from Alexandria Main University Hospital based on who was readily available, which means the results might not apply to other hospitals. Differences in workplace culture, hospital policies, or staff backgrounds at other institutions could lead to different outcomes.

### Data collection tool

The authors adopted the Patient Mobilization Attitudes and Beliefs Survey for the ICU (PMABS-ICU) questionnaire developed by Hoyer et al. [25]. This questionnaire has previously been published in the ward setting and the ICUs [25, 26]. The authors used the questionnaire in English, as CCNs and physicians are familiar with it.

PMABS-ICU is a self-reported questionnaire to assess perceived barriers to older adult patient mobilization among CCNs and physicians. This self-reported questionnaire consisted of twenty-six items divided into four items for knowledge, nine for attitudes, and thirteen for behaviors. Each statement had a five-point Likert scale: zero scored as not applicable; one scored as strongly disagree; two scored as disagree; three scored as neutral; four scored as agree; and five scored as strongly agree. The prior research calculated scores for the 26-item barrier scale and the three subscales: knowledge, attitude, and behavior [18, 26]. We contrasted the subscales (knowledge, attitude, and behavior) and overall scores, which ranged from 1 to 100 based on the respondents’ position; the highest score indicated the highest barriers [27]. This questionnaire is valid and reliable; the internal consistency of the overall scale and subscale was measured using the Cronbach α test (value 0.82), which was accepted [27]. 

This questionnaire was validated by five experts in the related field of Egyptian health care providers and was used without any changes due to the Creative Commons permission. The following statement is added to the heading of the questionnaire before participants submit their responses: “*Answer the following questionnaire statements considering the patient is older adults (aged > 65 years old)*, this age group is more likely to have functional deterioration and unfavorable outcomes after intensive care unit admission in the local healthcare system.” The scoring of negative items had been reversed before statistical analysis [25]. 

Additionally, CCNs and physicians’ demographic and job-related data were collected. This data encompassed age, gender, educational level, marital status, clinical position, ICU working experience, and ICU mobility protocol availability [28]. Also, the level of consciousness of older adult patients, caring for them, being on mechanical ventilation, and the number of invasive devices linked to them are all significant factors.

### Ethical consideration

The authors obtained ethical approval from the Faculty of Nursing, Alexandria University (IRB 00013620). They started collecting data after obtaining permission from the hospital authority. Participation in the research was voluntary, and participants had the right to agree or refuse to participate, depending on their decision. Their privacy was protected. The study’s execution was conducted while maintaining the confidentiality of the obtained data. The current study employed no invasive procedures or experimental protocols.

### Study procedure

**Fig. 1:**
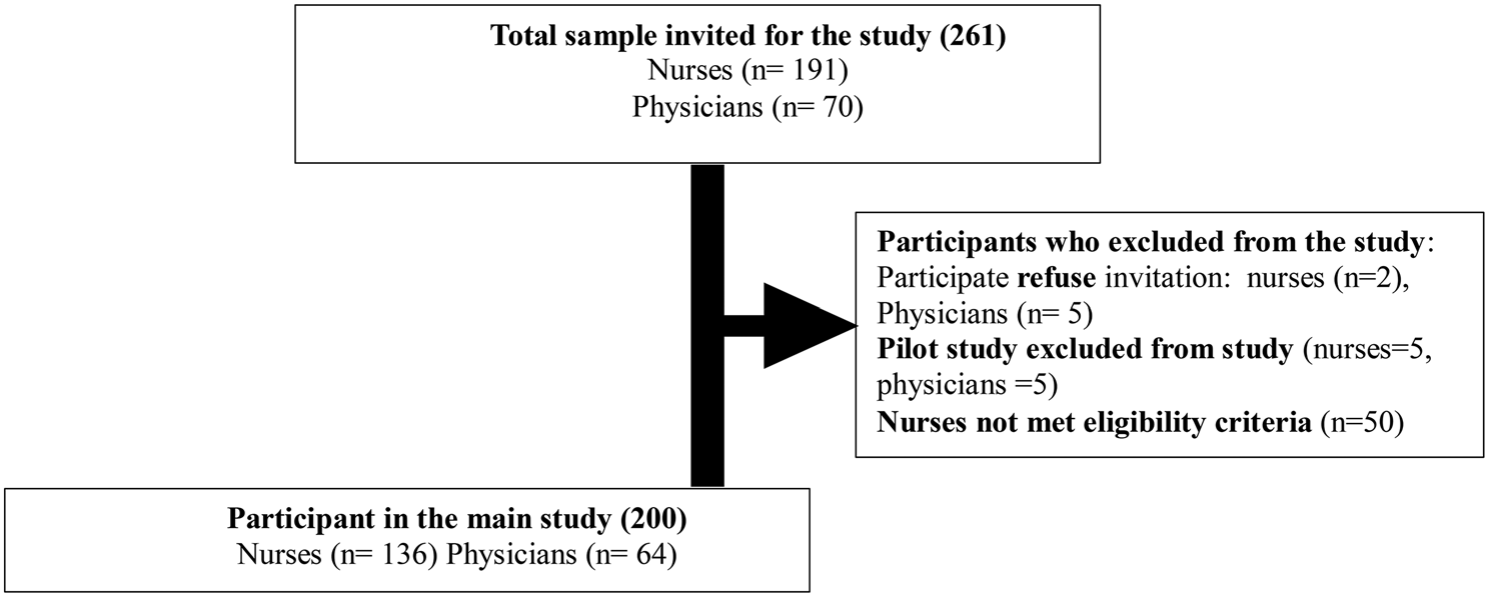
Sample flow chart

After obtaining ethical approval and hospital authority permission, the authors created a group on WhatsApp to invite participants from CCNs and physicians to join the study. Then, a Microsoft Form link was sent to this group. After finishing the submission, the participants had the right to leave the WhatsApp group. The response rate was 95%, and no missing data was found, as all questions were set as required. A pilot study assessed the tool’s applicability to five nurses and five physicians. A pilot study participant was excluded from the actual research. (Fig. 1) The study meticulously planned and tested the online questionnaire before its launch by CHERRIES’ requirements. The study’s goal, participants’ rights, and the need for confidentiality were thoroughly explained, allowing for easy informed consent. The findings were made more transparent and reliable since the recruitment procedure was well-documented, and technical measures were taken to prevent duplicate responses and guarantee secure data storage and processing [29].

### Statistical analysis

Data was downloaded in an Excel file from Microsoft Form and entered and coded in the SPSS program version 25. Demographic and job-related data are presented in numbers and means (standard deviation). The overall PMAB-ICU barrier scores and subscales were calculated based on the previous literature [18, 19]. We examined the distributions of overall and subscale scores using the mean (standard deviation). To evaluate differences between professions, we employed the independent sample t-test. Multiple linear regression was used to identify the factors that affect the overall scale and subscales for the PMABS-ICU questionnaire. A p-value of 0.05 or less was considered statistically significant for statistical analyses. The effect size was calculated using Cohen’s d for Independent Samples t Tests. (0.2 = Small effect; 0.5 = Medium; >0.8 = Large) [30]. 

## Results

Two hundred voluntary participants engaged in our study; one hundred and thirty-six were CCNs, and sixty-four were physicians. Only five physicians and two CCNs refused to participate in the study because they were uninterested. The completion rate was 95%, and no missing data was found because all questions were labeled “required” in the online questionnaire.

Table 1 illustrates the demographic and job-related characteristics of the participants. About 56.3% of physicians were aged between 40 and 50, and 78.7% of CCNs were aged between 20 and 30. Nearly 74.3% of CCNs were female, and 54.7% of physicians were male. Most CCNs and physicians had bachelor’s degrees (83.8% and 85.9%, respectively). More than half of CCNs (58.1%) were single, while 56.3% of physicians were married. The mean (SD) experience years for CCNS was 5.41(3.50), while 7.23(3.99) for physicians. Most CCNs and physicians confirmed the availability of mobility protocols in their working areas (83.1% and 73.4%, respectively). Most of the participants care for unconscious older adults (55.5%) and are attached to mechanical ventilators (94%). The mean (SD) of the number of connected devices for older adult patients was 3.34(1.24) for CCNs and 3.48(1.18) for physicians.

**Table 1:** Critical care nurses and physicians’ demographic and job-related characteristics

Demographic characteristics	Nurses (*n* = 136)		Physician (*n* = 64)	
	*N*	%	*N*	%
**Age**				
20-<30	107	78.7%	0	0.0%
30-<40	26	19.1%	28	43.8%
40-<50	3	2.2%	36	56.3%
**Gender**				
Male	35	25.7%	29	45.3%
Female	101	74.3%	35	54.7%
**Level of education**				
Bachelor	114	83.8%	55	85.9%
Postgraduate (master’s/PhD)	22	16.2%	9	14.1%
**Marital status**				
Single	80	58.1%	28	43.8%
Married	56	41.2%	36	56.3%
**ICU working experience years**				
Mean (SD)	5.41(3.50)		7.23(3.99)	
**Working with the ICU mobility protocol**				
Yes	113	83.1%	47	73.4%
No	23	16.9%	17	26.6%
**The level of consciousness of older adult patients**				
Consciousness	18	13.2%	8	12.5%
Semi-consciousness	48	35.3%	15	23.4%
Unconsciousness	70	51.5%	41	64.1%
**Caring for older adult patients on mechanical ventilation**				
Yes	126	92.6%	62	96.9%
No	10	7.4%	2	3.1%
**Number of attached invasive devices**				
Mean (SD)	3.34(1.24		3.48(1.18)	

Table 2 displays the overall and subscale PMABS-ICU scores for CCNs and physicians. The mean (SD) of the overall barrier score for critical care nurses was 113.16(16.7), significantly higher than the 107.75(10.9) reported by physicians (*P* = 0.007). This indicated that CCNs reported higher barriers than physicians. The effect size between CCNs and physicians on the Knowledge subscale is 0.484, indicating a moderate difference in knowledge scores between the two groups. The mean (SD) of attitude in CCNs was 26.7(55), which was of higher significance (*p* = 0.000) than physicians, 23.56(4.78). CCNs had a higher total behavior subscale 45.22 (11.20) than physicians 44.39 (4.79), with no statistically significant differences between them (*p* = 0.461). Among the three subcategories, fear of injury (behavior) was the highest among CCNs, 4.13(1.18), followed by increased work overload (attitude). The highest mean (SD) among the three subcategories in physicians was obtaining training about patient safety (knowledge subscale), 4.50 (92), followed by lack of time (behavior subscale), 4.430(81). The effect size on the Attitude subscale (0.647) indicates a moderate difference in perspectives between CCNs and physicians, while the small effect size on the Behavior subscale (0.086) suggests they behave similarly in this context. The effect size for the Total PMABS-ICU scores is 0.358, indicating a small difference between CCNs and physicians in their perceived or actual barriers to mobility.

**Table 2:** Overall and subscale PMABS-ICU scores for critical care nurses and physicians

Items	Nurses (*n* = 136)	Physician (*n* = 64)	t	*P* value
**Subscale 1: Knowledge subscale** ^**a**^	**Mean (SD)**	**Mean (SD)**		
I have received training on how to mobilize my patients safely.	**3.14 (1.38)**	**4.5(0.92)**		
I understand which patients are appropriate to refer to physiotherapy	**3.76 (1.23)**	**3.89 (1.169)**		
I understand which patients are appropriate to refer to Occupational Therapy.	**3.60 (1.23)**	**4.15 (1.12)**		
Unless contraindicated, I educate my patients to exercise or increase their physical activity while on my hospital unit.	**3.66 (1.28)**	**3.68 (1.28)**		
**Effect size (Cohen’s d)**	**0.484**			
**Total Knowledge score**	**14.44 (4.03)**	**16.23(2.87)**	**-3.191**	0.000*
**Subscale 2: Attitude subscale** ^**a**^				
My patients are too sick to be mobilized.	**2.23(1.062)**	**4.01(1.21)**		
Increasing my patients’ mobilization will harm them (e.g., falls, IV-line removal). ^c^	**3.39(1.43)**	**1.87(1.06)**		
A physiotherapist should be the primary care provider to mobilize my patients. ^c^	**2.20(1.40)**	**1.89(1.29)**		
Increasing the mobilization of my patients will mean more work for nurses. ^c^	**3.91(1.35)**	**2.10(1.40)**		
Increasing the mobilization of my patients will require more work from physio and/or occupational therapists. ^c^	**2.85(1.59)**	**2.39(1.52)**		
I believe that my patients mobilized at least once daily (if there is no contraindication) will have better outcomes. ^c^	**2.11 (1.40)**	**1.87(1.046)**		
I am not sure when it is safe to mobilize my patients.	**3.82 (1.30)**	**2.81(1.43)**		
I do not feel confident in my ability to mobilize my patients.	**3.77(1.36)**	**2.98(1.49)**		
My patients have time during their day to be mobilized at least once daily.	**2.44(1.41)**	**3.60(1.32)**		
**Effect size (Cohen’s d)**	**0.647**			
**Total Attitude score**	**26.75(5.00)**	**23.56(4.78)**	**4.328**	0.000*
**Subscale 3: Behavior subscale** ^**a**^				
We do not have the proper equipment and/or furnishings to mobilize my patients. ^c^	**3.66 (1.47)**	**4.21(1.06)**		
The physical functioning of my patients is regularly discussed between the patients’ healthcare providers	**3.41(1.38)**	**3.89(1.34)**		
Providers (nurses, physicians, physiotherapists, occupational therapists).	**3.16 (1.61)**	**3.73(1.48)**		
Nurse-to-patient staffing is adequate to mobilize patients on my unit. ^c^	**3.38 (2.18)**	**2.18(1.36)**		
My patients often have contraindications to be mobilized.	**3.38(1.39)**	**2.32(1.40)**		
Unless there is a contraindication, my patients are mobilized at least once daily by Nurses	**3.41(1.38)**	**4.23(1.01)**		
My leadership is very supportive of patient mobilization	**4.13(1.18)**	**4.23(1.08)**		
Increasing the frequency of mobilizing my patients increases my risk of injury	**3.38(1.39)**	**2.56(1.52)**		
Patients who can be mobilized usually have appropriate physician orders to do so.	**3.69(1.62)**	**4.09(1.26)**		
My patients are resistant to being mobilized ^c^	**3.62(1.63)**	**3.95(1.25)**		
Family members of my patients are frequently interested in helping mobilize them.	**3.42(1.37)**	**2.81(1.54)**		
I document the physical functioning status of my patients during my shift/workday. ^c^	**3.40(1.73)**	**4.43(0.81)**		
I do not have time to mobilize my patients during my shift/workday	**3.41(1.38)**	**1.73(1.06)**		
**Effect size (Cohen’s d)**	**0.086**			
**Total Behavior subscale**	**45.22(11.20)**	**44.39(4.79)**	**0.739**	**0.461**
**Overall**,** the PMABS-ICU scale is**^**a**^	**113.16(16.7)**	**107.75(10.95)**	**2.728**	0.007*
**Effect size (Cohen’s d)**	**0.358**			

^a^ Mean (SD) score for each question by position, score (1-5), where 1 = strongly disagree and 5 = strongly agree

b P value for student independent test < 0.05. c Response options were reverse-coded for subsequent analyses

0.2 = Small; 0.5 = Medium; 0.8 = Large

**Fig. 2:**
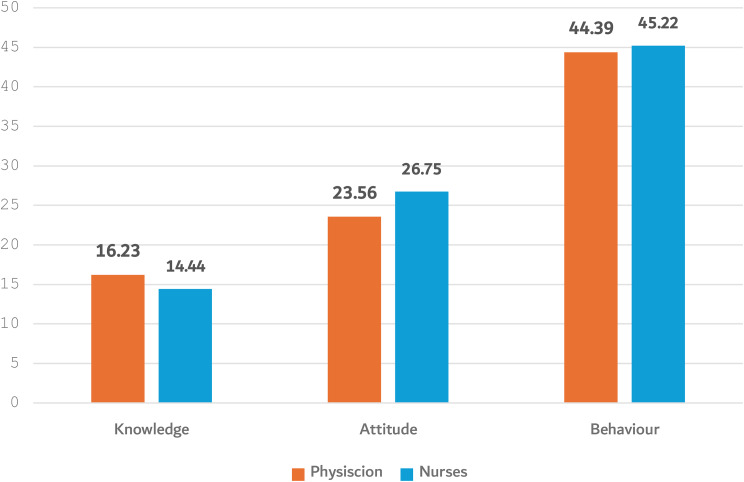
Comparison of the mean score of the subscale PMABS-ICU questionnaire between physicians and nurses

Figure 2 illustrates that the comparison between the total mean score of attitude and behavior subscale score PMABS-ICU questionnaire was marginally higher in nurses (26.75(5.00), 45.22(11.20), respectively) than in physicians (23.56(4.78), 44.39(4.79)). On the other hand, the mean score of knowledge in physicians (16.23(2.87)) was marginally higher than in nurses (14.44(4.03)). (Fig. 2)

Multiple linear regression analysis of demographic and job-related variables as predictors of knowledge, attitude, behavior, and overall scores shows that the model was significant (R2 = 0.220, F = 10.923, *p* < 0.001, R2 = 0.009, F = 0.372, *p* = 0.058, R2 = 0.134, F = 7.555, *p* < 0.001, and R2 = 0.128, F = 7.181, *p* < 0.001, respectively). A master’s degree was a factor of high knowledge, while the absence of protocol was a factor of low knowledge. Being a physician, having a master’s degree, and lacking a mobility protocol were factors of high attitude. Finally, being a physician and having a master’s degree were factors of high behavior, while the absence of protocol was a factor of low behavior (Table 3).

**Table 3:** Multiple regression analysis between the PMABS-ICU questionnaire about participant demographic and Job-Related data

Demographic characteristics	Total Knowledge subscale				Total attitude subscale				Total Behavior subscale				Overall scale			
	B	Beta	t	*p*	B	Beta	t	*p*	B	Beta	t	*p*	B	Beta	t	*p*
Position category (physician)	1.531	0.189	1.716	0.088	3.212	0.236	2.025*	0.044*	5.965	0.292	2.608*	0.010*	11.077	0.341	3.086*	0.002*
Age (40-<50)	0.010	0.022	0.201	0.841	-0.016	-0.020	-0.170	0.865	-0.038	-0.032	-0.286	0.775	-0.040	-0.021	-0.194	0.847
Level of education (master’s)	1.577	0.211	3.095	0.002*	2.253	0.200	1.893*	0.003*	2.923	0.023	2.093*	0.002*	1.923	0.200	2.893*	0.004*
Mobility protocol (Absence of protocol)	-3.321	-0.351	-5.245*	< 0.001*	3.923	0.200	2.893*	0.000*	-6.347	-0.267	-3.969*	< 0.001*	-9.345	-0.247	-2.723*	< 0.001*
Gender	0.011	0.006	0.092	0.927	0.056	0.039	0.530	0.597	0.066	0.055	0.780	0.436	0.045	0.038	0.542	0.588
	**R2 = 0.220**, F = 10.923*,***p*** < 0.001*				**R2 = 0.009**,** F = 0.372**,***p*** **= 0.058**				**R2 = 0.134**, F = 7.555*,***p*** < 0.001*				**R2 = 0.128**, F = 7.181*,***p*** < 0.001*			

F, p: f and p values for the model R^2^: Coefficient of determination B: Unstandardized Coefficients Beta: Standardized Coefficients t: t-test of significance *: Statistically significant at *p* ≤ 0.05

Table 4 illustrates the correlation matrix of the overall and subscale PMABS-ICU questionnaire. There was a moderate positive correlation between the overall score and subscale knowledge (*p* = 0.000), attitude (*p* = 0.000), and behavior (*p* = 0.000).

**Table 4:** Correlation matrix of the overall and subscale PMABS-ICU questionnaire

		Knowledge	Attitude	Behavior	Overall score
Knowledge	Pearson Correlation				
	Sig. (2-tailed)	**-**	**-**		**-**
Attitude	Pearson Correlation	-0.154^*^		-	
	Sig. (2-tailed)	0.029	-		
Behavior	Pearson Correlation	0.649^**^	-0.064	-	
	Sig. (2-tailed)	0.000	0.369		-
Overall score	Pearson Correlation	0.550^**^	0.592^**^	0.744^**^	
	Sig. (2-tailed)	0.000	0.000	0.000	-

* Correlation is significant at the 0.05 level (2-tailed). ** Correlation is significant at the 0.01 level (2-tailed)

## Discussion

Rehabilitation programs for older adult ICU patients must include mobilization. Unsurprisingly, over 20% of the older adult patients who could self-mobilize before leaving the medicine unit needed help walking upon discharge [31]. Healthcare providers’ attitudes and perspectives on older adult patients’ care, as well as institutional or unit policies, can pose challenges to the implementation of mobilization [32–34]. This challenges to mobilizing older adult patients in ICUs can be classified into patient-related, provider-related, and system-level factors, which inform the development of targeted initiatives to enhance early mobilization practices. Factors relating to older adult patients, such as old age, critical illness condition, patient contraindications for mobility, and patient fear from movement or fracture, that highlight the older adult patients’ behavioral barriers. Health care provider-related challenges, including insufficient training, fear of mobility catastrophic events, fear of increased risk for injury and apprehensions, underscore the necessity for continuous education and a culture transformation among healthcare providers. Systemic level problems, such as limited equipment, workplace culture, hospital policy, time constraints, and insufficient staffing, highlight the significance of institutional resources and support. Collaborative efforts among physicians, nurses, and allied personnel can mitigate various hurdles, thereby enhance early mobilization initiatives and eventually improve patient outcomes in ICU environments [35]. The overall score of the Patient Mobilization Attitudes And Beliefs Survey For The ICU PMABS-ICU questionnaire showed a significant difference between the perceived barriers for CCNs and physicians. CCNs, in contrast to physicians, had a more negative impression of the mobility barrier as a whole. These distinctions revealed a significant difference in the total score and the knowledge and attitude domains. While the behavior domain shows no significant difference in both groups, it was higher in CCNs than in physicians. Our findings align with Lewis et al. (2021), who reported that 92 nurses reported significantly higher perceived barriers to rehabilitation than nine physiotherapists [18]. These barriers included knowledge, attitudes, behaviors, and patient-specific factors. Similarly, Goodson et al. (2020) reported that 86 nurses perceived the highest overall perceived barrier score than 14 physicians [27]. 

In our study, CCNs frequently cited provider-related barriers such as the fear of causing injury more often than physicians. This might be partly because there are more females among the nurses than doctors. Research shows that female nurses often tend to be more emotionally careful when caring for patients, which might affect how they see risks and obstacles. Further research is recommended to examine the influence of gender on perceived barriers to mobility among geriatric patients, as gender-related differences may play a significant role in shaping mobility experiences and care outcomes. Since this study was conducted at just one Alexandria Main University Hospital, our results might not reflect what’s happening in other healthcare settings. System-level Factors such as hospital policies, workplace culture, and the mix of staff could have affected the differences we found between nurses and physicians. To get a more complete picture, future research should include multiple hospitals.

Regarding their level of knowledge, CCNs reported that they differ most from physicians (based on four questions). These questions emphasized the need for CCNs to get training and be aware of when to refer patients to physiotherapy or occupational therapy, as well as how to ensure patients’ safety. Other researchers have documented that nurses possess varying degrees of knowledge regarding the therapeutic domains of older adults’ patient care. These levels of knowledge have been found to range from low to average [2, 4, 36, 37]. 

Conversely, CCNS reported more obstacles with attitude and behavior subscale ratings than physicians. This shows that when it comes to older adult patients in particular, nurses are more worried about their mobility than physicians are. This dread impacts their perspective and conduct when caring for older adult patients. Increased workload, ensuring safety, and confidence during older adults’ patient mobilization may hinder older adults’ patient mobilization among CCNs according to patient-related, and provider-related factors. This finding aligns with Kim et al. [19] and Boehm et al. [38], who state that increased workload among studied participants was the highest barrier regarding early mobilization in ICUs.

From multi-regression analysis, the predictor model of position (physicians), age (40-<50), education level (master), and mobility protocol (absence) may be associated significantly with the overall score and three subscales. Also, the overall score showed a highly significant correlation between subscale knowledge, attitude, and behavior. The questionnaire score is not influenced by the varied years of experience of the participants studied. This finding aligns with Lewis et al. (2021), who reported that the overall score of the PMABS-ICU questionnaire was not associated with the years of clinical experience of the nurse studied [18]. Anekwe et al. [34] stated that most perceived barriers are affected by professional expertise and training, as reported by 138 participants. This finding was supported by Dagnachew et al. (2023), who noted that health professionals’ positive attitudes regarding early mobilization in the ICU were substantially correlated with their knowledge base and advocated for early mobilization within the ICU [21].

When examining the behaviors and attitudes of CCNs and physicians, it becomes apparent that older adult patients often refuse to leave their beds or express concerns about sustaining additional injuries. These factors were shown to be significant obstacles for CCNs. Besides, the confidence of CCNs in managing the mobility of older adult patients was a more substantial obstacle compared to physicians. Lack of sufficient equipment might pose a significant provider related barrier for CCNS and clinicians. A previous qualitative study by Brown et al. (2007) found that, compared to physicians (67%), 70% of nurses reported that older adult patients had mobility barriers. Weaknesses, ICU devices, and fear of falling were the top three patient-related obstacles, according to nurses [37]. Physicians reported that patients’ reasons and the existence of ambulatory equipment were the most significant obstacles. Also, Fontela et al. [8] reported that most of the studied participants, nurses, and physical therapists reported that the availability of time to mobilize ventilated patients and workload were the most reported barriers to early mobilization in the ICU. A Qualitative study by Gholamzadeh et al. [39] highlighted that healthcare providers may perceive negative stereotypes toward older adult patients. Age discrimination may hinder caring for older adult patients. The presence of age discrimination might impede the provision of care for older adult patients.

The identified gap between knowledge, attitudes, and behaviors among critical care nurses and physicians has significant implications for mobilizing older adult patients. While healthcare providers may possess adequate knowledge about the benefits of early mobilization, this knowledge does not always translate into positive attitudes or consistent clinical practice. Negative perceptions, fear of patient harm, and workload concerns may hinder the willingness to implement mobilization strategies. Moreover, inconsistent behavior among healthcare providers can create confusion within the multidisciplinary team, leading to fragmented care [40]. Addressing this gap is essential to improve patient outcomes, enhance teamwork, and ensure that evidence-based practices are reliably and systematically applied in critical care settings.

Several studies by Cooper et al. (2021), Najjar et al. (2021), and Young et al. (2018) have examined many possible barriers, including organizational factors like staffing levels and equipment availability, as well as patient-related factors like medical problems and sedative requirements. In addition to challenges connected to the organization, barriers to effective mobilization policy in healthcare include workload and poor training of healthcare providers, as well as environmental factors such as the physical layout of the unit and safety concerns. The study sought to elucidate the challenges encountered by physicians and CCNs in promoting mobility among this specific group of patients by identifying these obstacles [41]. For older adult patients, interdisciplinary teams are crucial in determining the advantages and barriers perceived by engaging in mobility activities. The quality of life for older adult patients can be significantly enhanced by addressing the issues that arise from immobility and promoting early and safe mobilization [42, 43]. Although this study found several vital obstacles to patient mobilization, the results were not divided into areas linked to patients, personnel, or organizations. This restricts how detailed possible interventions may be. Structured barrier categorization models should be used in future studies to allow for more focused and situation-specific solutions.

### Conclusion and study implications

This study has shown that most CCNs reported a more pronounced perception of barriers regarding the mobility of older adult patients than physicians. They may improve patient outcomes by removing these obstacles, including lowering the risk of consequences from immobility and encouraging functional independence.

In addition, develop and implement evidence-based training programs focusing on the importance of safety, and techniques of early mobilization for older adult patients. These programs should be tailored to nurses and physicians, using case-based learning, simulations, and interdisciplinary workshops. Embed mobility planning into multidisciplinary rounds, ensuring that CCNs and physicians jointly set and review daily mobility targets for each geriatric patient. Also, updated rounding templates, team communication strategies, and shared accountability for CCNs and physicians.

Our study shows a real need to support healthcare staff in helping patients stay mobile. This includes assessing and improving the availability of assistive devices (e.g., mobile lifts, walkers, low beds) and designating mobility aids or physiotherapy support for peak ICU hours. This could mean offering training to help CCNs feel more confident and less afraid of causing injury or giving physicians better tools and strategies to manage their time and communicate more effectively. These practical steps could make it easier to include mobility support in patient care, showing how our findings can lead to meaningful changes in everyday practice.

### Study limitations

One limitation of the study is the use of an English-language questionnaire, which may have excluded participants such as technical staff or nursing aides who are not proficient in English. Although English is the official language of communication in the study setting, this language restriction may have led to the underrepresentation of culturally and linguistically diverse groups, potentially omitting important perspectives relevant to the research topic.

A further limitation of this study is the use of WhatsApp as the primary method for participant recruitment, which may have introduced response bias. Individuals without access to smartphones or those lacking familiarity with digital communication platforms were likely underrepresented in the sample. While the study focused on the perceptions of healthcare professionals, it did not include the perspectives of patients themselves and family members regarding mobility barriers. The absence of direct patient input limits the comprehensiveness of the findings, particularly in understanding patient-experienced barriers and needs about mobility within the healthcare environment.

The small sample size limited the generalization of the study findings to other settings or populations. Further studies integrating randomized sampling are recommended, using a large sample for more generalization. The current study lacks clarity regarding obstacles to patients, staff, and the organization.

The study’s inability to distinguish between organizational, personnel, and patient-related constraints is one of its limitations. This kind of barrier classification would improve the results’ interpretability and actionability. The viewpoints of healthcare professionals were the exclusive focus of this study; older adults' opinions were not considered. Consequently, it offers a biased perspective on the obstacles to mobilization.

This study is limited by its single-center, non-representative sample, which restricts generalizability. Differences in age and experience between nurses and physicians may have influenced their responses. Future research with more diverse, multi-center samples is recommended to confirm these findings.

## Data Availability

Under inquiry for scientific studies, the correspondent researcher will share researchable information.
